# The Potential of Using Bisr Date Powder as a Novel Ingredient in Biscuits Made of Wheat Flour Only or Mixed with Barley

**DOI:** 10.3390/foods13121940

**Published:** 2024-06-19

**Authors:** Haiam O. Elkatry, Sukainah E. H. Almubarak, Heba I. Mohamed, Khaled M. A. Ramadan, Abdelrahman R. Ahmed

**Affiliations:** 1 Food and Nutrition Science Department, Agricultural Science and Food, King Faisal University, Al-Ahsa 31982, Saudi Arabia; suainah-essa@hotmail.com (S.E.H.A.); arahmed@kfu.edu.sa (A.R.A.); 2 Home Economics Department, Faculty of Specific Education, Ain Shams University, Abassia, Cairo 11772, Egypt; 3 Biological and Geological Sciences Department, Faculty of Education, Ain Shams University, Cairo 11341, Egypt; 4 Central Laboratories, Department of Chemistry, King Faisal University, Al-Ahsa 31982, Saudi Arabia; kramadan@kfu.edu.sa; 5 Department of Agricultural Biochemistry, Faculty of Agriculture, Ain Shams University, Hadayek Shobra, Cairo 11241, Egypt

**Keywords:** antioxidants, bisr Al-Khalas, biscuit, chemical analysis, flavonoids, phenolic, physical properties, organoleptic properties

## Abstract

An overproducing date fruit with limited industrial utilization leads to significant waste and losses, especially in the early stage of date maturity known as bisr. This study aimed to investigate the potential use of bisr date powder (BDP) at different concentrations (25%, 50%, and 100%) as a natural sweetener instead of sugar and barley flour as a source of dietary fiber, vitamins, and minerals instead of wheat flour (50%) in biscuit production over storage periods of 7, 14, and 21 days. The analysis revealed that the bisr Al-Khalas powder sample had a moisture content of 11.84%, ash content of 2.30%, and crude fiber content of 10.20%. Additionally, it had a low protein (2.50%) and fat (0.77%) content, with total carbohydrates at 82.59%. The gradual substitution of bisr Al-Khalas in biscuit production resulted in an increased moisture, ash, fat, protein, crude fiber, and iron content, as well as a decrease in total carbohydrate percentage. A chemical analysis of bisr Al-Khalas powder demonstrated high levels of antioxidants, with 248.49 mg gallic acid/g of phenolic compounds, 31.03 mg quercetin/g of flavonoids, and an antioxidant activity ranging from 42.30%, as shown by the DPPH test. The peroxide content was 0.009 mg equivalent/kg. Biscuit samples with different proportions of bisr Al-Khalas showed an improved resistance to oxidation compared to samples without bisr Al-Khalas, with increased resistance as the percentage of replacement increased during storage. Physical properties such as the diameter, height, and spread percentage, as well as organoleptic properties like color, flavor, aroma, and taste, were significantly enhanced with higher levels of bisr Al-Khalas in the mixture. Biscuit samples fortified with 100% pure bisr Al-Khalas powder were found to be less acceptable, while samples with a 25% substitution did not negatively impact sensory properties. In addition, acrylamide and hydroxymethylfurfural (HMF) were not detected in bisr powder and biscuit samples prepared at different concentrations (25%, 50%, and 100%). In conclusion, the study suggests that bisr Al-Khalas powder, an underutilized waste product, has the potential to add value to commercial biscuit production due to its high nutritional value and extended storage period resulting from its potent antioxidant activity.

## 1. Introduction

Bakery products are among the most widely consumed foods, in large amounts on a daily basis, and have an important role in human nutrition. The fortification of bakery products with functional ingredients in order to improve their health properties and nutritional profile is a commonly used approach to fulfill current consumer demands [1]. The principal ingredient of bakery products is wheat flour, which is responsible for the structure and bulk of the product, followed by water, yeast, salt, and other ingredients. However, the increasing demand for foods with additional health benefits has promoted the inclusion of bioactive compounds or dietary fibers as new ingredients, representing one of the most researched and used trends in recent years [2].

Biscuits are popular baked goods with low final water content (1–5 g/100 g) and, typically, three main ingredients (flour, sugar, and fat), as they are high in sugar and have low amounts of antioxidants, fiber, and minerals [3]. Since they have a variety of tastes, a long shelf life, widespread acceptance, and practicality, and are relatively inexpensive, biscuits are a common food item that wide spectrums of people eat [4]. In response to the market competition and the consumer demand for natural, healthy products, biscuits’ nutritional composition is being altered to improve their nutritious value and functionality. In this sense, widely consumed biscuits could function as a useful vehicle for the transfer of bioactive compounds. Biscuits are now receiving more attention for their potential to create functional foods since they can be fortified [5].

Because of their busy lifestyles, people tend to eat more ready-to-eat, less healthful foods, which can raise their chance of contracting diseases including diabetes, obesity, cardiovascular disease, and high blood pressure. Therefore, adding dietary fiber and bioactive substances to baked goods can help reduce the negative health effects of bad eating habits [6]. In the case of fiber, it is well-known that the population does not consume sufficient amounts of fiber on a daily basis, so the enrichment of bakery products with dietary fiber could help to increase the recommended fiber intake. Likewise, the incorporation of fiber reduces blood glucose levels due to digestible carbohydrates, making it possible for patients with diabetes to consume bread or bakery products [6]. Date bioactive compounds have the potential to serve as a natural substitute for synthetic antioxidants like butylated hydroxyanisole (BHA) and butylated hydroxytoluene (BHT), hence reducing toxicity levels and associated processing costs [7].

Nowadays, the valorization of byproducts from the food industry by reincorporating them into the food chain is one of the main approaches used to improve the sustainability of food production through the reduction of the amount of waste generated, thus promoting the circular economy [8,9,10,11]. Using dates and their byproducts to enhance the nutritional and functional quality of bread and bakery goods has become more popular in recent decades. Because date fruit and its byproducts naturally contain a variety of groups of phytochemicals, the scientific world is beginning to take notice of the date fruit and its byproduct’s appropriateness for use in health-promoting functional food composition [12].

Dates are considered essential crops and part of the global human diet. They can be commonly taken at three phases of development: bisr, rutab, and tamer [13,14]. Nowadays, the majority of date fruits are consumed as fresh or dried pitted dates at the tamer stage (containing 10–30% moisture), with small amounts consumed at the rutab or ripe stage (containing 30–35% moisture), and minimal amounts consumed at the firm and crunchy bisr stage or the first stage of dates’ maturity (containing 50% moisture) [15].

Due to the present restricted processing capacity, date overproduction results in severe losses, particularly for dates with high moisture content, such as bisr dates. Even though the bisr stage (dropping from palms) contains all the nutrients that dates possess after full maturity, its annual loss amounts to 15–20% of date production, which is either deemed an underutilized quantity or used to make animal feed [16]. Because it has a low glycemic index and low simple sugar content, this stage is a good sugar substitute [17]. Hence, using the bisr stage as a date with high fiber content and a unique functional component in the food industry is sustainable [18].

The consumption of dates contributes to overall health due to their richness in bio-nutrients, such as dietary fibers, proteins, carbohydrates, low fat, and minerals, with carbohydrates being the most abundant, averaging an estimated 50–89 g of total sugars per 100 g of date fruits [14]. In addition, dates have been found to include vitamins like thiamine, cobalamin, riboflavin, retinol, pyridoxine, and ascorbic acid, as well as carotene, flavonoids, and anthocyanins that affect date color [14]. Because dates include bioactive components including carotenoids, phenols, sterols, anthocyanins, and antioxidants that enhance their functional and health features, they have the potential to treat gastrointestinal and cardiovascular disorders [17].

Numerous fruits and their byproducts are utilized as ingredients to add fiber and other bioactive substances or to prolong the shelf life of baked goods [1]. In addition, Alsenaien et al. [17] described that bisr date powder (BDP) is considered an agricultural byproduct and can be used as a novel food ingredient. Bisr date powder (BDP) is rich in dietary fiber, flavonoids, and phenolic and antioxidant compounds, and can be used as a natural sweetener instead of sugar. This study aimed to investigate the potential use of bisr date powder (BDP) at different concentrations (25%, 50%, and 100%) as a natural sweetener instead of sugar and barley flour as a source of dietary fiber, vitamins, and minerals instead of wheat flour (50%) in biscuit production over storage periods of 7, 14, and 21 days on their chemical, sensory, and physical properties, as well as their content of iron, antioxidant activity, acrylamide, and hydroxymethylfurfural (HMF). The current study investigated increasing BDP levels rather than using sugars in biscuit production.

## 2. Materials and Methods

### 2.1. Preparation of Date Powder

**Figure 1:**
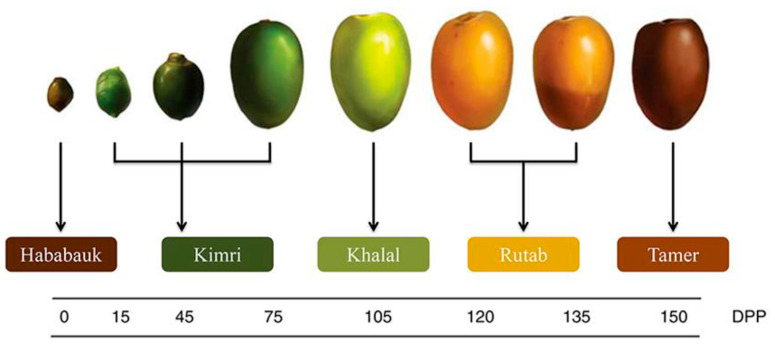
Growth stages of date fruits [16]: Bisr (Khalal) stage.

The National Research Centre for Date Palm, Al Ahsa, Saudi Arabia, provided fresh dates at the Bisr stage (*Phoenix dactylifera* L.) (Khalas variety, production season 1441 H, 2020) (Figure 1). Fruits were de-pitted, cut into small pieces, and dried in a vacuum-drying oven (OV-11, Jeio Tech Co., Ltd., Seoul, Republic of Korea) at 75 ± 1 °C for 24 h, and crushed in grinder (M-20, IKA-Werke, GMBH & CO. KG, Staufen, Germany) for 2 min. The obtained date powder was sifted in a steel mesh sifter (0.85 mm openings) to obtain fine homogenized particles. The obtained powder was stored in a close container at 4–5 °C until use.

### 2.2. Biscuit Components and Chemical Compounds

The biscuits were made using materials that were purchased from the Al Ahsa, Saudi Arabia local market. The components were sucrose (commercial grade), fat (butter), fresh whole egg, baking powder (sodium bicarbonate and cream of tartar), and vanilla (pure vanilla). Soft wheat flour (72% extraction) was obtained from Mills Company in Hufuf, Saudi Arabia. Sigma–Aldrich (Sigma, Livonia, MI, USA) provided all analytical grade compounds that were used.

### 2.3. Biscuit Preparations

The raw materials used to manufacture the biscuits were weighed and prepared: 147 g sugar, 200 g butter, 14 g vanilla, 120 g eggs, and 8 g baking powder (sodium bicarbonate). The fat was mixed with sugar; then, we added eggs, vanilla, and 600 g of flour. The recipe followed a traditional creaming process. First, softened butter and powdered sugar were creamed together until light and fluffy, mimicking the texture of mayonnaise. Then, dry ingredients like flour, baking powder, and salt were gradually incorporated. A small amount of water was added to achieve a crumbly dough consistency. This dough was chilled for an hour, before being rolled into a sheet and cut into uniform circles (diameter: 50 mm; weight of each dough piece: 15 g) using a cookie cutter. Baking was carried out using traditional oven convection at 180 °C for 10 min, then left to cool to room temperature. After that, it was packed in tightly sealed containers and stored until sensory evaluation and chemical tests were carried out. This is for the first control sample, which consists of wheat flour alone. The second control sample consists of wheat and barley flour with a mixing ratio of 50:50 for each. The experimental design was a randomized complete block design. All treatments have three replications. Table 1 shows the percentages of substituting bisr powder instead of sugar in biscuit samples.

### 2.4. Sensory Evaluation of Biscuit Samples

The test was carried out following the rules of the Declaration of Helsinki [19]. Twelve individuals, ages 25 to 50, participated in the sensory evaluation panel. They were chosen from among postgraduate students, lab assistants, senior researchers, and staff members of King Faisal University’s College of Agricultural and Food Sciences’ Food and Nutrition Sciences Department. Although the individuals who participated were not trained, they were given written and verbal explanations about the evaluation processes before the biscuit evaluation. Biscuit samples were evaluated in two separate sessions in a well-lit and ventilated sensory room with an average temperature of 25 °C. During each session, biscuit samples labeled with three-digit randomized codes were served, and the panelists were asked to perform visual and ortho- and retro-nasal evaluations of the samples for the attributes of smell, color, taste, flavor, and overall acceptability of the samples after they were randomly assigned codes [20]. The panelists rated these attributes based on a six-point hedonic scale. The panelists used water between samples to eliminate the residual taste according to the AACC 33-50.02 method [21].

### 2.5. Spread Ratio Measurement of Biscuit Samples

A Vanier caliper (WA-VC115 Wiika, Beijing, China) was used to measure the thickness and diameter at two different places, and the average was calculated. The spread ratio was then computed using the Kaur et al. [22] technique, which involved dividing the average biscuit diameter by the average biscuit thickness after the biscuits had cooled for 30 min:

(1) $$\mathrm{Spread}\;\mathrm{ratio}=\frac{Average\;diameter\;(cm)}{Average\;thickness\;(cm)}\;$$

### 2.6. Proximate Composition Analysis

#### 2.6.1. Estimation of Moisture Content

The Ahn et al. [23] method of oven-drying at 100 °C for 24 h resulted in a consistent weight, which was used to calculate the moisture content. The following formula was used to estimate the percentage of moisture:

(2) $$\mathrm{Moisture}\;\%=\frac{Weight\;of\;sample\;before\;drying-Weight\;of\;sample\;after\;drying}{Sample\;weight\;(g)}\times 100$$

#### 2.6.2. Estimation of Ash Content

Sample’s ash content was determined using Ahn et al. [23] methodology. In a traditional muffle furnace, 2 g of ground sample was dried for 5–6 h at 600 °C. After weighing the prepared sample and placing it in the furnace, the residue was also weighed. Using the following formula, the ash content was determined:

(3) $$\mathrm{Ash}\;\%=\frac{Weight\;of\;crucible\;with\;dried\;sample-Weight\;of\;empty\;crucible}{Weight\;of\;crucible\;with\;raw\;sample-Weight\;of\;empty\;crucible}\times 100$$

#### 2.6.3. Estimation of Fat Content

The total fat content of the sample was determined using an automated Soxhlet method according to the method of AOAC [24]. Three grams of the sample were taken and dried in a drying oven to stabilize the weight at a temperature of 70 °C for a period of 30 min. The samples were then placed in a Soxhlet device using the solvent ethanol for 16 h, and the solvent was separated using a rotary evaporator. Fat was then estimated using the following equation:

(4) $$\mathrm{Fat}\;\%=\frac{Weight\;of\;crucible\;after\;extraction-Weight\;of\;crucible\;before\;extraction}{Weight\;of\;sample}\times 100$$

#### 2.6.4. Estimation of Protein Content

The Kjeldahl technique was used to estimate the protein content of the samples [25]. After weighing one gram of the prepared sample, it was put into a Kjeldahl digestion flask along with 0.5 g of copper sulphate and about 15 g of potassium sulphate. Sulfuric acid (20 mL) was then added to help the material break down. After being gradually heated to 400 °C for 1.5 h, the flask was allowed to cool. Subsequently, 50 mL of sodium hydroxide (40%) and roughly 75 mL of deionized water were added and properly mixed. The tube was then placed into the digesting unit, and, after around six minutes of distillation, the desired sample was recovered and placed in a beaker with a solution of 4% boric acid and a mixed indicator. The solution was then titrated using 0.1 HCl. Using the following formula, the protein content was determined:

(5) $$N\%=\frac{The\;titration\;volumes\;of\;the\;sample-The\;titration\;volumes\;of\;the\;blank\times The\;concentration\;of\;HCl\;(Normality)\;\times 14.007}{The\;weight\;of\;the\;sample\;(g)}\times 100$$

The molecular weight of nitrogen (n) is 14.007.

Following the calculation of the nitrogen concentration, the following equation was used to convert it to a protein content using the appropriate (6.25) conversion factor:

Percentage (%) of protein = N × 6.25 (6)

#### 2.6.5. Estimation of Carbohydrate Content

Carbohydrates were estimated using the AOAC [26] method according to the equation:

Crude carbohydrate content (%) = 100 − {Moisture content (%) + Crude protein content (%) + Crude fat content (%) + Crude ash content (%)}(7)

#### 2.6.6. Estimation of Crude Fiber Content

Crude fiber is defined as the amount of dry residue that remains after a sample is digested under certain conditions with a solution of 1.25% sulfuric acid and 1.25% sodium hydroxide. Then, 200 mL of 1.25% sulfuric acid was added to a sample weighing 5 g that was free of fat and moisture. After 30 min of boiling, the solution was filtered using a Buchner funnel and other filtration equipment. After being thoroughly cleaned with water to remove any remaining acid, the residue was moved into a beaker. Then, 200 mL of sodium hydroxide (1.25%) was added, and the mixture was once more heated for 30 min. After another filtering and water wash, the remnants were removed. After being moved to the pre-weighted crucible, the residues were baked in a hot air oven at 100 °C until they reached a consistent weight. After that, the residue was burned in a muffle furnace, and a weight reduction was seen [27]. The following formula was used to obtain the crude fiber content:

(8) $$\mathrm{Crude}\;\mathrm{fiber}\;\%=\frac{\left(Weight\;of\;residue-Weight\;of\;ash\;after\;ignition\right)}{Weight\;of\;sample}\times 100$$

### 2.7. Preparation of the Sample Extract of Samples of Biscuits Made from Wheat Flour Alone or Mixed with Barley Flour and Fortified with Bisr Date Powder in Different Proportions

According to the method of Majzoobi et al. [28], one gram of sample powder was mixed in 20 mL of 95% methanol, and then we left the mixture for an hour in a dark place, after which the mixture was filtered and the residue was extracted with 95% methanol. We then kept the extract in a cool place until it was ready to be used.

#### 2.7.1. Determination of Total Phenol Content

Folin–Ciocalteu reagent was used to determine the total phenol content (TPC), with some modifications as described in Singleton and Rossi [29]. The procedure involved diluting 20 µL of the sample in 180 µL deionized water, mixing it with 200 µL of the reagent, vortexing it, adding 800 µL of sodium carbonate, and then incubating for 30 min at 40 °C. The optical density was measured at 765 nm. The TPC concentrations were determined using the standard curve of gallic acid, which covered the range of 0–100 µg/mL.

#### 2.7.2. Determination of Flavonoids

According to Zhishen et al. [30], flavonoids were determined using the aluminium chloride colorimetric method. Then, 150 µL of the sample, 150 µL methanol, and 75 µL 5% NaNO_2_ were combined in a vial, and the mixture was left to react for five minutes at room temperature. After an additional five minutes, 1.25 mL of 7% AlCl_3_ and 0.5 mL of 5% NaOH were added, and the absorbance at 510 nm was determined. The flavonoid concentrations were determined using the quercetin standard curve in the range of 25–200 µg/mL.

#### 2.7.3. Determination of Antioxidant Activity

The sample was then processed into powder using an electric grinder after being dried for 30 min at 70 °C in a drying oven. Utilizing the 2,2-Diphenyl-1-picrylhydrazyl (DPPH) free radical scavenging method [31], the extracts’ capacity to scavenge free radicals was ascertained. Two grams of the sample extract were weighed and added to a beaker with 10 mL of methanol in order to prepare it. After that, it was agitated for four hours with a magnetic stirrer to aid in extraction. Whatman filter paper No. 42 was then used to filter the extracted materials. A new DPPH solution containing 0.002% was made in methanol, and its absorbance was measured at 517 nm. After adding 50 μL of the pure extract to a 3 mL DPPH solution, the mixture was left to stand in the dark for 15 min. At 517 nm, the absorbance was once more measured:

(9) $$\mathrm{DPPH}\;\mathrm{scavenging}\;\mathrm{capacity}\;(\%)=\frac{\left(A\;control-A\;sample\right)}{A\;control}\times 100$$

A = absorbance at 517 nm

### 2.8. Determination of Peroxide Value of Samples of Biscuits Made from Wheat Flour Alone or Mixed with Barley Flour and Fortified with Bisr Date Powder in Different Proportions

The peroxide value was estimated according to the method [26]. The liberated iodine was titrated using sodium thiosulfate, so the blank sample was without oil; the oil sample was used after extraction. From the samples, there were 5 mL of a mixture (2: acetic acid + 3: chlorophore), potassium iodide, 5 mL of distilled water, and drops of starch solution. The oil samples were mixed with a mixture of acetic acid and chloroform, then shaken and added to 5 mL of distilled water with drops of potassium iodide. The color changed to blue, and then it was titrated with sodium thiosulfate and shaken until it changed from a blue solution to a colorless solution. The peroxide value was expressed as mg equivalent/kg and was determined by the following equation:

(10) $$\mathrm{Peroxide}\;\mathrm{value}=\frac{Sodium\;thiosulfate\;titrated\times (Blank\;(ml)\;titration\;volume-Sample\;(ml)\;titration\;volume)}{Weight\;of\;sample\;(g)}\times 1000$$

The standard of sodium thiosulfate (0.01 M) = 0.3 molar

1000 = conversion factor g/kg

### 2.9. Determination of Iron Content of Bisr Date Powder (BDP) and Samples of Biscuits Made from Wheat Flour Alone or Mixed with Barley Flour and Fortified with Bisr Date Powder in Different Proportions

Potassium thiocyanate was used to determine iron content using the AOAC [26] method. Then, 1 mL 30% H_2_SO_4_, 1.0 mL potassium persulfate solution, and 1.5 mL 40% KCNS solution were added to water to make up 6.5 mL of the mineral solution. After 20 min, a red color appeared and the optical density was read at 540 nm.

### 2.10. Determination of Acrylamide of Bisr Date Powder (BDP) and Samples of Biscuits Made from Wheat Flour Alone Fortified with Bisr Date Powder in Different Proportions

Samples were extracted to acetic acid (0.2 mM) water solution and pre-extracted to ethyl acetate to avoid the negative impact of salts in chromatography system according to Ciesarov’a et al. [32]. The sample preparation was as follows: 10,000× *g* of a homogenized sample was weighed into a 10 mL centrifuge tube with a cap, then 50 μL of the internal standard solution (0.0020 g of acrylamide-D3 in 100 mL of water) and 9 mL of acetic acid (0.2 mM) were added. After shaking by a vortex mixer for 30 s, the mixture was sonicated for 5 min. Then, 500 μL of Carrez solution I (15 g of K_4_[Fe(CN)6]∙3H_2_O in 100 mL of water) and 500 μL of Carrez solution II (30 g of ZnSO_4_.7∙H_2_O in 100 mL of water) were added and mixed for 1 min. After that, the mixture was centrifuged (Hermann, Northeim, Germany) at 9000 rpm for 10 min at −5 °C. A volume of 5 mL of the clear supernatant was transferred to a separator funnel; 5 mL of ethyl acetate was added and mixed well. The ethyl acetate layer was removed, and the extraction step was repeated twice with 5 mL of ethyl acetate. Then, 3 × 5 mL of ethyl acetate layers were collected and evaporated in a vacuum rotatory evaporator at 35 °C to dryness. The residue was dissolved in 1 mL of acetic acid solution (0.2 mM) and filtered through a 0.45 μm pore size nylon syringe filter (Q-Max RR Syringe Filters, Frisenette ApS, Knebel, Denmark). LC-MS analysis was performed with a HPLC system 1260 Infinity II series (Agilent Technologies, Santa Clara, CA, USA) coupled to an Agilent 6160 MSD equipped with an ESI interface. The analytical separation was performed on Zorbax C18 (100 mm, 3 μm) column (Agilent, USA) using an isocratic mixture of 5 mL of methanol, 1 mL of acetic acid, and 500 mL of deionized water at a flow rate of 0.5 mL/min at 25 °C. The ESI mass spectrometry detection was performed in a positive ESI + mode with drying gas (N2) flow 8 L/min and 350 °C temperature, nebulizer pressure 50 psi, capillary voltage 2.5 kV, and sheath gas flow 11 L/min at 250 °C. Data acquisition was performed using single-ion monitoring (SIM) with 72 for acrylamide. The quantification of acrylamide was calculated with a calibration curve of the standard compound in the range of 10 to 200 µg/mL. Time of analysis was 10 min; retention time of acrylamide was 0.432 min. LOD of the applied procedure was 25 μg/L; LOQ was 40 μg/L.

### 2.11. Determination of HMF of Bisr Date Powder (BDP) and Samples of Biscuits Made from Wheat Flour Alone Fortified with Bisr Date Powder in Different Proportions

HMF was extracted by the mixture composed of methanol and water in a ratio 80:20 (*v*/*v*). Approximately 1.0 g of homogeneous sample was mixed with 10 mL of extraction mixture and sonicated for 5 min. After filtration through 0.45 μm pore size nylon syringe filters (Frisenette ApS, Knebel, Denmark), samples were ready for injection. The Agilent 1260 HPLC-MS system (Agilent Technologies, Santa Clara, CA, USA) at flow rate 0.6 mL/min, an autosampler and MSD equipped with an ESI interface were used for separation and quantification of HMF in samples. Data acquisition was performed using single-ion monitoring (SIM) with 127 and 91 *m*/*z* ions for HMF. Separation was performed on a Zorbax C18 SB column (150 × 4.6 mm, 5 μm; Agilent Technologies, USA). The isocratic mobile phase consisted of 95% of 0.5 formic acid, and 5% of 0.1 formic in acetonitrile. Time of analysis was 20 min; retention time of HMF was 11.0 min. An internal standard of 150 µg/mL of methyl benzoate was used for calibration.

### 2.12. Statistical Analysis

The results were expressed as mean ± standard deviation (SD). The data obtained from the analysis were subjected to one-way and two-way analysis of variances. The Duncan Multiple Range Test (DMRT) was used to distinguish between all means at a 5% probability level (*p* = 0.05).

## 3. Results and Discussion

### 3.1. Chemical Composition

The chemical composition of bisr date powder (BDP) is presented in Table 2. The results indicated a wide variation in date bisr chemical composition. The bisr powder had moisture (11.84%), ash (2.30%), low percentages of protein (2.50%) and fat (0.77%), while, had high percentages of crude fiber (10.20%) and total carbohydrates (82.59%). These findings are consistent with those of Alqahtan et al. [33]. In addition to their health advantages, dietary fibers contain many useful features that are crucial for food preparation, like their capacity to retain water, swell, enhance viscosity, and gel [33]. Therefore, the utilization of date fruits with high fiber content at the bisr stage as a functional ingredient in bakery products will be beneficial in improving their properties as well as their sensory characteristics. Dates are usually used as ingredients in food and bakery preparations to provide a good taste to the final products [17].

Furthermore, the chemical composition of biscuit samples made from wheat flour alone or mixed with barley flour enriched with varying concentrations of bisr date powder (25, 50, and 100% as a sugar substitute) was investigated. It was observed from the results in Table 2 that the moisture content of the biscuits increased significantly with the increase in replacing sugar with bisr date powder. The highest moisture content was detected in biscuits prepared by replacing sugar with 100% bisr date powder (10.51%) and biscuits prepared with 50% wheat flour and 50% barley flour, replacing the sugar with 100% bisr date powder (9.10%), as compared to the control. It could be noticed that the moisture content increased with the increase in the bisr date powder substitution. The high content of moisture is due to the high sugar content of the bisr date which binds water in fortified biscuits. These results are consistent with those of Dhankhar et al., [34], who studied the effect of adding chickpea flour and date powder to refined wheat flour in the production of biscuits. The mixture with high concentrations of date powder has the highest percentage of moisture (60%). In addition, the increase in moisture content in the Khalas dates sample can be attributed to the formation of hydrogen bonds with water molecules, which is associated with the higher hydroxyl group content in bisr date than sugar. These result in reduced free water movement and an increase in the moisture content of the prepared Kleicha [35].

The biscuit samples showed significant increases in the ash and fiber content with the increasing rate of addition of bisr date powder. The relatively high ash and fiber content in the fortified samples was also noted with bisr date powder and replaced with barley flour due to the higher mineral content of barley flour compared to wheat flour. The highest ash and fiber content was detected in biscuits prepared by replacing sugar with 100% bisr date powder (1.17%, and 19.8%) and biscuits prepared with 50% wheat flour and 50% barley flour, replacing the sugar with 100% bisr date powder (1.63%, and 44.6%), respectively, as compared to the control.

High ash content is an indication of a high mineral content. Date seeds are found to be rich in minerals [3], and an improved mineral content was observed in composite bakery products in a previous study [36]. Therefore, the addition of higher amounts of date seed powder may increase the ash content and, hence, the mineral content of composite cookies [3]. Similar results were found by Bader Ul Ain et al. [37], who found that biscuit samples enriched with bisr date powder and barley flour had increased fiber levels. Wheat flour biscuits have large amounts of both soluble (13.32%) and insoluble dietary fiber (8.79%). According to El-Sharnouby et al. [38], bisr date powder contains a significant percentage of dietary fiber (10.20%).

Biscuits fortified with barley flour and bisr date powder can be considered a good source of fiber. Dietary fiber is characterized by its healthy properties, such as improving the human digestive process and lowering the glycemic index. The chemical composition of biscuit samples with different replacement levels indicates that they have greater nutritional value than biscuits made with flour and 100% wheat. These results showed an increase in the nutritional value of biscuits substituted with different concentrations of bisr date and are consistent with those found in other studies that worked on integrating various products like sweet chickpeas, date powder, corn flour, sugar beet, chickpea and date flour, and potato powder in biscuits [34,39,40].

The biscuit sample produced with 50% wheat flour, 50% barley flour, and 100% bisr date powder as a substitute for sugar had the highest fat content (7.57%) when compared to the sample control. These results are in accordance with the results of Ikuomola et al. [41], who found that the fat content in biscuits prepared with wheat flour and barley flour was 29.86–32.36%. The increase in fat content in biscuit samples may be due to the partial replacement of barley flour due to the high fat content in barley flour. The increased fat content of biscuits contributes to their higher flavor and texture. In addition, fats are a rich source of energy and necessary as carriers of vitamins soluble in fat (A, D, E, and K) [41].

The higher protein content in the fortified samples can also be attributed to the increased concentration of barley flour and bisr date powder. The decrease in the percentage of protein in the control sample may also be due to the high concentration of protein and gluten in flour. These findings are in accordance with the study of Ibrahim et al. [42], who found that the nutritional value of biscuits increased with the addition of different concentrations of date paste (0%, 10%, and 40%) to the flour. It was observed that the percentage of protein in the samples increased with an increase in the replacement ratio of the date paste powder.

The fortification of biscuit samples with different levels of bisr date powder as a sugar substitute resulted in a decrease in the total carbohydrates in fortified biscuit samples with an increasing percentage of barley flour replacement and bisr date powder. These results obtained are consistent with the results of the study by Dhankhar et al. [34], who aimed to improve the properties of biscuits by adding chickpea flour and date powder to refined wheat flour in different proportions. A decrease in total carbohydrates was observed with an increase in the replacement percentage of bisr date powder in the biscuit samples. Therefore, bisr date powder can be used as a sweetening agent instead of sugar. These results differ from the results of Amin et al., [43], who aimed to study the effect of adding different concentrations of date powder (5%, 10%, 20%, and 40%) as a natural sweetener instead of sugar as a natural sweetener on the chemical composition, physical properties, and sensory properties of the biscuits. The results showed that increasing the rate of replacement of date powder led to an increase in total carbohydrates in biscuit samples.

In addition, increasing the concentration of date powder increased the protein, fat, ash, fiber, and moisture levels to their maximum values in cake fortified with 100% date powder. The effect of date powder on carbohydrate content showed a decreasing trend at a low substitution level (25%) and an increasing trend at a high concentration of date powder. The increase in protein, fat, ash, fiber, and moisture in sponge cake following the date powder addition could be attributed to the high amounts of these chemical attributes in date powder [44]. The incorporation of date powder as replacer of sugar led to a decrease in carbohydrates with an increase in the substitution ratio [45]. Similarly, previous studies reported that incorporating date powder into bakery products increased the protein, fat, ash, fiber, and moisture content [46,47].

### 3.2. Antioxidant Activity

#### 3.2.1. Phenolic Compounds

Table 3 shows the phenolic content of manufactured biscuit samples using wheat flour, either alone or mixed with 50% barley flour fortified with bisr date powder in different proportions. The bisr date powder sample had a total phenol content of 248.49 mg gallic acid/g, and this value is close to that reached by Umar Nasir et al. [48], who found that the phenolic value in dried dates was 239.5 mg equivalent gallic acid/g. The substitution of bisr date powder instead of sugar in the biscuit samples makes a noticeable difference from the control sample. The phenolic content in biscuit samples significantly increased with an increasing sugar replacement ratio with bisr date powder and replacing wheat flour with barley flour. A previous study proves the positive correlation between the antioxidant capacity and total phenol concentration in dates [49]. Therefore, having dates means having good phytochemicals that are very useful for human health, such as decreasing levels of cholesterol, and a decreasing risk of heart disease, and cancer [50,51].

The biscuits prepared with 100% wheat flour and 50% wheat and 50% barley flour with 100% bisr date powder had the highest values of phenolic content of 252.20 and 256.38 mg gallic acid/g, respectively. The results showed that, during the different storage periods at 7, 14, and 21 days and at a temperature of 4 °C, there was a slight decrease in the total phenol content in the sample of biscuits (Table 3). The most pronounced increases in total phenol content were detected in biscuits prepared with 50% wheat flour and 50% barley flour, and replacing sugar with 100% bisr date powder during the different storage periods compared to the control sample. The results of this study showed that the phenols in biscuits produced from wheat and barley flour and fortified with bisr date powder were more stable at 4 °C than in biscuits made from wheat flour and fortified with bisr date powder. This indicates that biscuits fortified with bisr date powder have a high content of phenolic compounds and have high antioxidant activity. According to Najjar et al. [3], date seed powder has high levels of antioxidants. It can also be used as a functional ingredient in the food industry to improve the quality of baked goods, as evidenced by the improved antioxidant properties of composite cookies with increasing substitution levels. According to our findings, adding date seed powder instead of flour greatly increased the amount of polyphenols and flavonoids, as well as the antioxidant activity.

#### 3.2.2. Flavonoids

Flavonoids are natural, biologically active compounds that have a high ability to prevent diseases including cancer and cardiovascular diseases. These benefits are attributed to its antifungal activity, antioxidants, and flavonoids, which are also powerful anti-inflammatory agents [52]. In addition to their nutritional value, dates are rich in flavonoids that have antioxidant activity [53].

**Figure 2:**
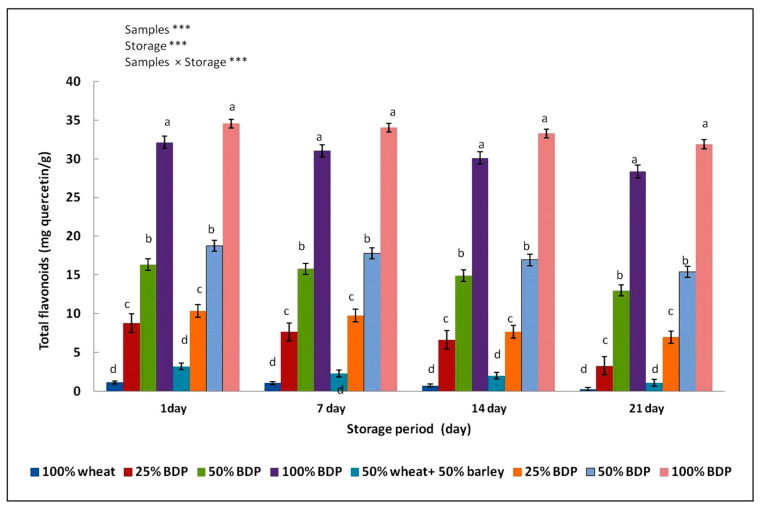
Total flavonoid content (mg quercetin acid/g) of biscuit samples made from wheat flour alone or mixed with barley flour and fortified with bisr date powder in different concentrations. The mean ± SD of three replicates is used to represent the data. Duncan’s test at *p* < 0.05 indicates that the various letters denote the significance within each bar. *** Highly significant difference at *p* < 0.05.

The results in Figure 2 showed that the flavonoid content of bisr date powder and biscuit samples were produced using wheat flour, either alone or mixed with 50% barley flour, replacing sugar powder with bisr date powder in different proportions. The bisr date powder sample had a total flavonoid content of 31.03 mg quercetin/g, and this value is close to the result of the study of Allouche et al. [54], who found that the total flavonoid content in different varieties of dates grown in Tunisia was about 12.10–26.09 mg quercetin/g.

A variation in flavonoid values was observed between the control sample and the biscuit samples in which the sugar was replaced with bisr date powder. The flavonoid content increased in biscuit samples with an increase in the percentage of replacing sugar with bisr date powder and replacing wheat flour with wheat and barley flour. A sample of biscuits prepared with 100% wheat and 50% wheat flour and 50% barley flour and a replacement with 100% bisr date powder has the highest value of flavonoid content of about 32.15 and 34.55 mg quercetin/g, respectively.

The results showed that, during the storage period of 7, 14, and 21 days at a temperature of 4 °C, there was a significant decrease in the total flavonoid content in the sample of biscuits. The results of this study showed that the flavonoid content in biscuits made from 50% wheat flour and 50% barley flour and with bisr date powder was more stable at 4 °C than in samples of biscuits made from wheat flour and sugar. These results indicate that the flavonoid content in bisr date powder was the main contributor to its antioxidant activity. Flavonoids and polyphenols both significantly improve human health. While flavonoids have the capacity to prevent low-density lipoproteins from oxidation and to interfere with the generation and spread of free radicals, polyphenols are recognized for their antioxidant, anti-inflammatory, and anticarcinogenic properties [3]. The findings suggest that bisr date powder is a useful tool for increasing biscuits’ antioxidant capacity.

#### 3.2.3. Antioxidant Activity Test (DPPH)

**Figure 3:**
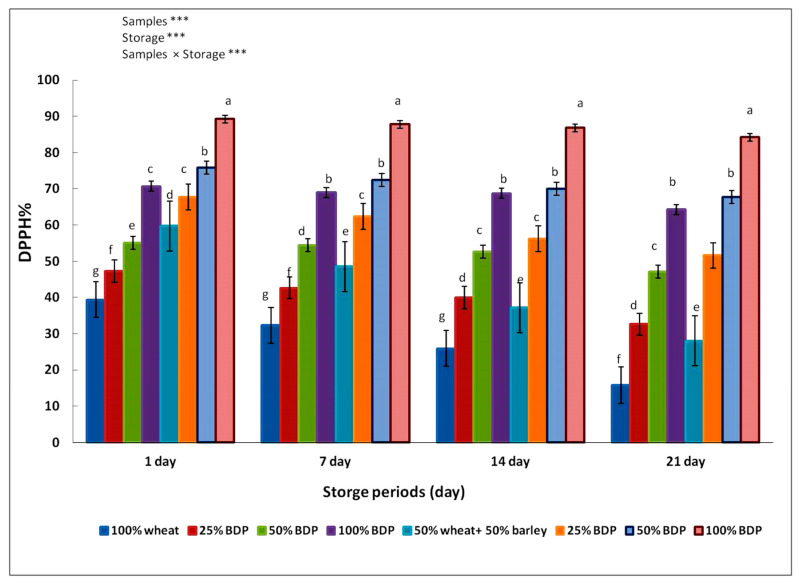
The antioxidant activity (DPPH%) of biscuit samples made from wheat flour alone or mixed with barley flour and fortified with bisr date powder (BDP) in different proportions. The mean ± SD of three replicates is used to represent the data. Duncan’s test at *p* < 0.05 indicates that the various letters denote the significance within each bar. *** Highly significant difference at *p* < 0.05.

The data in Figure 3 show the antioxidant activity of biscuit samples made by using wheat flour, either alone or mixed with 50% barley flour, and different concentrations of bisr date powder. The DPPH value for the bisr date powder sample was 42.30%. It is also noted that there is a significant difference between the control sample and the biscuit samples in which sugar was replaced with bisr date powder. This may be due to the amount of antioxidant compounds in the bisr date powder. The antioxidant content in the biscuit samples increased with an increase in the sugar replacement ratio with bisr date powder and the replacement of wheat flour with barley flour. It showed that the biscuits prepared with 50% wheat and 50% barley flour, replacing sugar with 100% bisr date powder, had the highest antioxidant value of 89.23%. The results were shown during the storage period of 7, 14, and 21 days at a temperature of 4 °C. 

The results in the present study showed that the antioxidant activity observed in biscuit samples made from 50% wheat, 50% barley flour, and bisr date powder was higher and more stable at 4 °C compared to biscuit samples made from wheat flour and sugar. The reason can be attributed to the antioxidant compounds of the bisr date powder, such as ascorbic acid, vitamin E, carotenoids, and selenium, as well as to flavonoids and other phenolic components [54]. This is consistent with Amira et al. [55], who studied the antioxidant activity of four varieties of Tunisian dates in three stages of maturity (Bisr, Rutab, and Tamer). The results showed that all samples in the “Bisr” stage obtained the highest value of antioxidants. This indicates that the bisr date powder has high antioxidant activity. 

Due to its high antioxidant content and the improved antioxidant properties of composite biscuits with increasing replacement levels, bisr date powder can be used by the food industry as a functional ingredient to improve the quality of baked goods. These findings are in line with those of Najjar et al. [3], who discovered that date seeds have the potential to be used as a natural, active raw material for food applications such as in baked goods. According to Al-Farsi et al. [56], the higher the phenolic content of the seeds, the stronger the antioxidant activity when compared to the other byproducts of dates. Mistrello et al. [57] and Al Juhaimi et al. [58] observed strong antioxidant activity in Sukkary date seed powder. These results imply that the powder also contributes to the high antioxidant activity of bakery goods.

#### 3.2.4. Peroxide Value (PV)

The peroxidase value indicates the amount of peroxide compounds in food, which measures oxidation products. This occurs during the storage of food and shows the level of rancidity in fats and oils during storage. This is responsible for the undesirable flavor and odor in the manufactured products [59].

**Figure 4:**
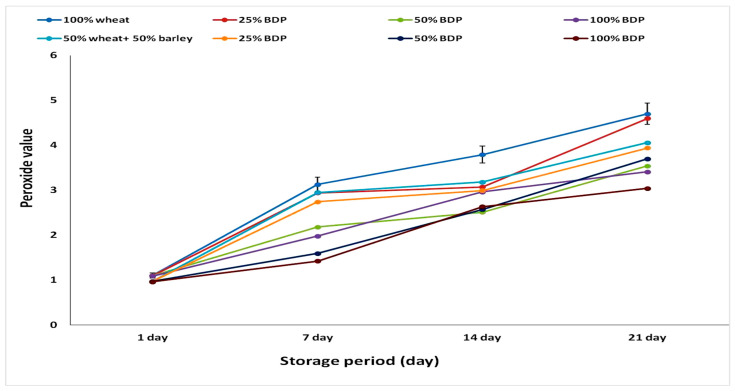
Peroxide content (mEq/kg) of samples of biscuits made from wheat or barley flour using bisr date powder (BDP) in different proportions during the storage period. The data are represented as the mean ± SD of three replicates.

Figure 4 and Table 4 show the peroxide value of biscuits produced using wheat flour, either alone or mixed with 50% barley flour, in which the sugar is replaced with bisr date powder in different proportions. According to the results obtained, the peroxide value of bisr date powder was 0.009 mEq/kg. The peroxide value of the biscuits prepared with 100% wheat flour increased by about 1.10, 3.13, 3.79, and 4.70 mEq/kg with the increase in storage days at 1, 7, 14, and 21 days, respectively, while the biscuits prepared with 50% wheat flour and 50% barley flour recorded peroxide values of about 0.97, 2.95, 3.18, and 4.06 mEq/kg at storage days of 1, 7, 14, and 21 days, respectively.

The value of peroxide is demonstrated to be significantly decreased as compared to control samples when bisr date powder is substituted for sugar in different concentrations in biscuits produced with wheat flour alone or in combination with 50% barley flour. During all storage days (1, 7, 14, and 21 days) at 4 °C, the biscuits made with 100% wheat flour and 50% wheat flour + 50% barley flour with 100% bisr date powder had the lowest levels of peroxide, indicating the significant role of antioxidants produced from the addition of bisr date powder to biscuit samples. The results of this study are consistent with the results of the study conducted by Al-Alawi et al. [60], who found that tree palm fruits are natural products and have therapeutic properties. Numerous investigations into the effects of watery date fruit extract (Degles nour variety) on the protection against oxidative damage and hepatotoxicity resulting from subchronic exposure to dimethoate on mice’s livers have reported varying results. However, the data presented here indicate that the extract was able to repair liver damage [60].

### 3.3. Iron Content

Bisr date powder is an important and rich source of essential elements such as iron, with a content of about 5.14 mg/100 g. The results indicated that the iron content of the biscuit samples increased significantly with an increase in the replacement ratio of bisr date powder in 100% wheat flour and 50% wheat flour with 50% barley flour as compared to control samples (Table 5). The control samples (100% wheat flour and 50% wheat flour with 50% barley flour) had the lowest concentrations of iron, about 2.37 and 5.17 mg/100 g, respectively. In contrast, the biscuit sample produced from 50% wheat flour and 50% barley flour with 100% bisr date powder had the highest value of iron content, reaching 9.36 mg/100 g. These increases in iron content in biscuits containing bisr dates are due to the fact that wheat flour also contains high concentrations of iron. These results are in accordance with El-Gammal et al. [61], who reported that Fe and Zn in wheat flour reached about 2.54 and 0.23 mg/100 g, respectively. The nutritional composition of biscuits can vary depending on the type of flour used. Alebiosu et al. [62], also reported an improvement in the iron content (3.73 mg/100 g–6.50 mg/100 g) of cookies produced from wheat–sorghum–defatted coconut composite flour. Iron is an essential trace element which plays vital roles such as hemoglobin formation and the oxidation of fats, protein, and carbohydrates [63]. The WHO [64]’s daily recommended intake of iron for children (6–59 months) is 5.8 mg/100 g. Hence, children can acquire the required iron from consuming samples together with other foods.

The results of this study are consistent with the findings of Ibrahim et al. [42], who manufacture biscuits with different percentages of date powder (10%, 20%, and 40%) as a substitute for flour date. The results showed that the higher the replacement rates for date powder, the greater the iron content in the manufactured biscuit samples. It can be concluded that adding bisr date powder to biscuit samples led to an improvement in the iron content of biscuits, which is beneficial for people who suffer from anemia. It has been proven to improve hemoglobin levels [42].

### 3.4. Sensory Evaluation

The data in Table 6 show the sensory evaluation values of biscuit samples manufactured using wheat flour alone or mixed with 50% barley flour, in which the sugar is replaced with bisr date powder in different proportions on the color, odor, taste, flavor, and overall acceptability. A sensory analysis of the newly formulated biscuits is crucial, as any sensory characteristic of food can significantly impact consumer choices and the recommendation for the marketplace based on its nutritional value and overall acceptability [65].

A sample of biscuits prepared with 50% wheat flour and 50% barley flour and sugar was replaced with 100% bisr powder, which had the best acceptance rate in terms of color, which may be due to an increase in the percentage of adding sugar to 100%, giving the biscuit samples a dark color during baking. This may be due to a change in color due to an increase in the proportion of wheat flour replacing barley flour, which provides more protein for the Maillard reaction to occur, which is often desired in baked goods. These findings are consistent with studies by Najjar et al. [3], Gómez and Martinez [66], Aksoylu et al. [67], and Ashoush and Gadallah [68] that documented changes in biscuit color brought about by the addition of byproducts such as seeds. As a result, the addition of date seed powder greatly changed the color of the cookies; the composite cookies had a darker hue, which is due to the inherent dark pigmentation of all date seeds, independent of variety. We cannot conclude from the color analysis results that the panelists are exclusively interested in light-colored products because some products, such as the Khalas and Zahidi variants, scored higher than others. Interestingly, when compared to zero- or low-level samples, date seed composite cookies with high substitutions are sometimes preferred [3].

In terms of odor and flavor, their acceptance significantly increases with the increase in the percentage of replacing sugar with bisr date powder, as well as with an increase in the percentage of replacing wheat flour with barley powder, which is a positive point for the addition. They expressed it as a new addition with a distinctive flavor that has high acceptability in terms of smell and flavor, which may be due to barley proteins, which are responsible for the palatable flavor. All the sensory parameters (appearance, surface, interior colors, texture, aroma, and taste with their overall acceptability) were the best with the biscuit samples supplemented by 10% date fruit powder [51].

As for taste, all samples obtained similar values, but it is noted that the prepared biscuit sample by replacing sugar with 50% bisr date powder gave the best rating, and the reason may be that substituting with this ratio led to a balance in the sweet taste of the biscuits as a result of the high sugar content. Of the sugar fructose and glucose, the sample of biscuits prepared with 50% and 50% wheat flour obtained from barley flour and replacing the sugar with 25% bisr date powder had the highest degree of acceptance in the overall acceptability compared to other samples.

The results of the study are consistent with a study by Amin et al. [43], who addressed the quality attributes of fortified biscuits with date powder at a rate of 5, 10, 20, and 40%. Samples of biscuits fortified with date powder were obtained (5.10%), with the highest preference in smell, taste, flavor, color and overall acceptability compared to biscuits with 100% sugar (control). It also agrees with the results of a study by Ibrahim et al. [42], who worked to develop and evaluate the nutritional content of date biscuits as a functional food. The replacement rates were 10%, 20%, and 40%, where the sample that was replaced with 20% date powder had the highest degree of acceptance with the control sample, while increasing the replacement percentage led to lower values of overall acceptability of biscuits, as this addition significantly affected the texture of the biscuits when the percentage increased, which, in turn, increased the hardness of the biscuits. The results indicate that it is possible to replace sugar with powdered sugar by up to 50% in biscuits made with wheat flour and 50% barley flour without affecting the sensory quality of the resulting biscuits.

Furthermore, probably, the impact of date powder on the sensory characteristics of sponge cake arises from the existence of certain components in the powder that influence the color (protein and carbohydrates) via the Millard reaction, the taste resulting from the phenolic compounds in the powder, and the overall acceptance owing to the remaining components in the powder [17,33].

### 3.5. Physical Properties

Biscuit spread is defined as the ratio of the diameter to thickness and is one of the important tests conducted on biscuits. A larger biscuit diameter and a higher spreading ratio are quality attributes in biscuit manufacturing [17].

**Figure 5:**
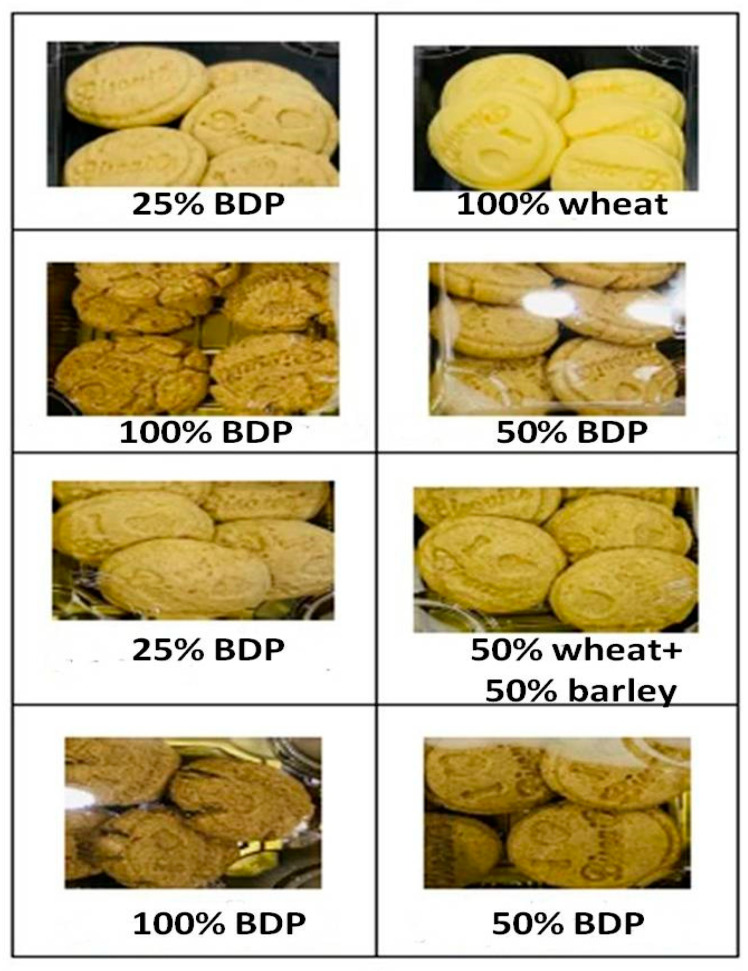
The biscuits prepared by addition of bisr date powder.

Table 7 and Figure 5 show the physical properties of manufactured biscuit samples using wheat flour, either alone or mixed with 50% barley flour, replacing sugar with bisr date powder in different proportions. The results obtained showed that the spread ratio (%) of the control sample for biscuits made with 100% wheat flour and sugar was 9.60%; increasing the bisr date powder led to the spread ratio (%) being significantly increased. Similar findings revealed that people prefer cookies with a higher spread ratio [69]. Additionally, according to Handa et al. [69], bigger cookie diameters and higher spread ratios are desired qualities.

In our present study, the height of the biscuits significantly increased with increasing bisr date powder concentrations. The maximum spread ratio (10.86%) was detected in biscuits made with 50% wheat flour and 50% barley flour with 100% bisr date powder. The reason may be due to replacing 50% of wheat flour with barley flour, which weakened the gluten network, which leads to a decrease in gluten, which is an undesirable characteristic in cookies, as well as causing the top surface to crack, which is a desirable characteristic in cookies [70]. Therefore, it is clear that replacing wheat flour with barley bran led to an increase in the spread ratio, which improved the quality characteristics of biscuits.

The findings of this investigation align with the findings of Karra et al. [71], who explained that the results of the physical properties of biscuits fortified with date powder were in different percentages (3, 6, and 9%). Increasing the percentage of wheat flour replaced with tamer powder caused an increase in the spread ratio. Similar results were also observed in the study conducted by Ikuomola et al. [41], who found that samples of biscuits made from wheat flour and barley bran in different proportions increased as the spread ratio of the samples increased with the increase in the percentage of replacing wheat flour with barley bran, and it ranged between 7.97% and 8.68%.

### 3.6. Acrylamide and Hydroxymethylfurfural (HMF)

**Figure 6:**
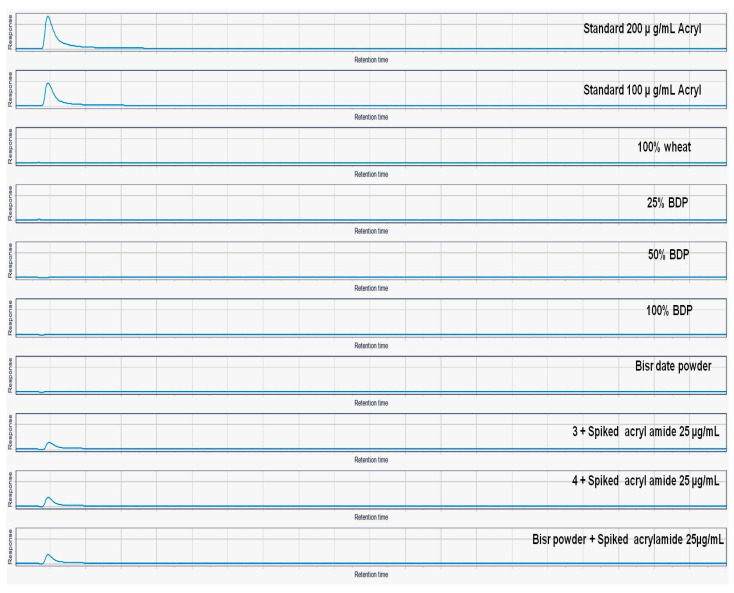
LC-MS TIC of detection of acrylamide in bisr powder and biscuit samples prepared with wheat flour and sugar replaced by different concentrations of bisr date powder (25, 50, 75, and 100%).

**Figure 7:**
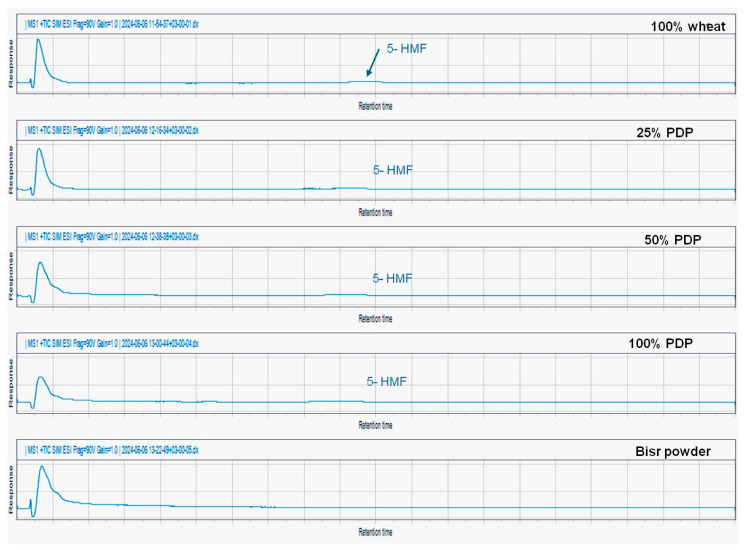
LC-MS TIC of detection of HMF in bisr powder and biscuit samples prepared with wheat flour and sugar replaced by different concentrations of bisr date powder (25, 50, 75, and 100%).

The results in Figure 6 and Figure 7 showed that there was no detection of acrylamide and hydroxymethylfurfural (HMF) in bisr date powder and biscuit samples prepared with wheat flour and sugar replaced by different concentrations of bisr date powder (25, 50, 75, and 100%).

## 4. Conclusions

Few studies are performed on using bisr date powder in the production of bakery goods. Therefore, the idea of the research was based on the use of bisr date powder in the production of biscuits, by its substitution with sugar in different proportions of 25, 50, 75, and 100%, made from wheat flour only or a mixture of wheat and barley flour by 50% each to obtain a product rich in fiber. Our results show that the substitution of sugar with bisr date powder significantly upgraded the content of polyphenols and flavonoids, as well as the antioxidant activity. The biscuits manufactured from bisr date powder have acceptable sensory properties, and this addition also contributed to slowing down the process of rancidity and extending the shelf life of biscuits. Because of their high fiber content, these biscuits may be a nutritionally suitable choice for diabetics. The incorporation of bisr date powder in food formulations will help to cover the current consumer demands for foods made with ingredients of natural origin, with high nutritional value, and with health properties beyond the merely nutritional without any formation of acrylamide and hydroxymethylfurfural.

## Figures and Tables

**Table 1:** The materials used in the formulation of biscuit with the substitution of sugar with different concentrations (0, 25, 50, and 100%) of bisr date powder (BDP).

Samples	Wheat Flour %	Barley Flour	Sugar %	Bisr Powder %
100% wheat	100	0	100	0
25% BDP	100	0	75	25
50% BDP	100	0	50	50
100% BDP	100	0	0	100
50% wheat + 50% barley	50	50	100	0
25% BDP	50	50	75	25
50% BDP	50	50	50	50
100% BDP	50	50	0	100

**Table 2:** The chemical composition (%) of bisr date powder (BDP) and samples of biscuits made from wheat flour alone or mixed with barley flour and fortified with bisr date powder in different proportions.

Treatment	Moisture	Ash	Protein	Fat	Raw Fiber	Carbohydrate
**Bisr date powder (BDP)**	11.84 ± 0.00	2.30 ± 0.00	2.50 ± 0.02	0.77 ± 0.01	10.20 ± 0.01	82.59 ± 0.00
**100% wheat**	7.56 ± 0.17 ^e^	0.60 ± 0.01 ^g^	10.50 ± 0.03 ^g^	3.60 ± 0.00 ^d^	7.30 ± 0.00 ^f^	77.73 ± 0.01 ^a^
**25% BDP**	8.22 ± 0.92 ^d^	0.74 ± 0.01 ^f^	11.25 ± 0.03 ^f^	3.90 ± 0.00 ^d^	12.90 ± 0.01 ^e^	75.88 ± 0.02 ^b^
**50% BDP**	9.87 ± 0.98 ^b^	0.98 ± 0.02 ^e^	11.95 ± 0.02 ^e^	4.20 ± 0.01 ^cd^	14.40 ± 0.02 ^d^	72.98 ± 0.02 ^c^
**100% BDP**	10.51 ± 0.68 ^a^	1.17 ± 0.02 ^d^	12.10 ± 0.01 ^e^	4.33 ± 0.01 ^c^	19.80 ± 0.00 ^b^	71.87 ± 0.01 ^d^
**50% wheat + 50% barley**	6.60 ± 0.13 ^g^	1.38 ± 0.01 ^c^	13.40 ± 0.02 ^d^	7.02 ± 0.030 ^b^	8.12 ± 0.00 ^f^	73.67 ± 0.01 ^c^
**25% BDP**	7.20 ± 0.00 ^f^	1.49 ± 0.02 ^b^	13.99 ± 0.01 ^c^	7.25 ± 0.26 ^b^	16.90 ± 0.01 ^c^	70.07 ± 0.02 ^e^
**50% BDP**	8.39 ± 0.00 ^d^	1.52 ± 0.02 ^b^	14.71 ± 0.03 ^b^	7.30 ± 0.16 ^ab^	19.90 ± 0.00 ^b^	68.40 ± 0.01 ^f^
**100% BDP**	9.10 ± 0.18 ^c^	1.63 ± 0.03 ^a^	16.82 ± 0.03 ^a^	7.57 ± 0.23 ^a^	24.60 ± 0.30 ^a^	65.88 ± 0.01 ^g^
**Samples**	***					
**Bisr powder**	***					
**Samples × Bisr powder**	***					

The mean ± SD of three replicates is used to represent the data. Duncan’s test at *p* < 0.05 indicates that the various letters denote the significance within each column. *** Highly significant difference at *p* < 0.05.

**Table 3:** Total phenol content (mg gallic acid/g) of biscuit samples made from wheat flour alone or mixed with barley flour and fortified with bisr date powder in different concentrations.

Treatment	1 Day	7 Days	14 Days	21 Days
**100% wheat**	7.14 ± 0.1 ^d^	5.20 ± 0.2 ^d^	4.01 ± 0.2 ^d^	2.70 ± 0.1 ^d^
**25% BDP**	69.14 ± 0.6 ^c^	66.39 ± 0.7 ^c^	59.34 ± 0.8 ^c^	44.28 ± 0.5 ^c^
**50% BDP**	131.10 ± 0.8 ^b^	129.18 ± 0.8 ^b^	124.22 ± 0.9 ^b^	112.32 ± 0.6 ^b^
**100% BDP**	252.20 ± 1.2 ^a^	250.62 ± 1.2 ^a^	235.77 ± 1.5 ^a^	229.41 ± 1.2 ^a^
**50% wheat + 50% barley**	8.19 ± 0.5 ^d^	6.75 ± 0.2 ^d^	4.9 ± 0.1 ^d^	3.70 ± 0.1 ^d^
**25% BDP**	70.23 ± 1.0 ^c^	67.22 ± 0.8 ^c^	61.05 ± 0.8 ^c^	54.30 ± 0.6 ^c^
**50% BDP**	133.19 ± 1.3 ^b^	131.60 ± 0.9 ^b^	126.14 ± 1.0 ^b^	116.05 ± 0.9 ^b^
**100% BDP**	256.38 ± 1.2 ^a^	254.50 ± 1.2 ^a^	250.04 ± 1.3 ^a^	241.60 ± 1.4 ^a^
**Samples**	***			
**Storage**	***			
**Samples × Storage**	***			

The mean ± SD of three replicates is used to represent the data. Duncan’s test at *p* < 0.05 indicates that the various letters denote the significance within each column. *** Highly significant difference at *p* < 0.05.

**Table 4:** Peroxide content (mEq/kg) of biscuit samples made from wheat flour alone or mixed with barley flour and fortified with bisr date powder in different concentrations.

Treatment	1 Day	7 Days	14 Days	21 Days
**BDP**	0.009 ± 0.00	0.009 ± 0.00	0.009 ± 0.00	0.009 ± 0.00
**100% wheat**	1.099 ± 0.00 ^a^	3.13 ± 0.00 ^a^	3.79 ± 0.00 ^a^	4.70 ± 0.00 ^a^
**25% BDP**	1.095 ± 0.01 ^b^	2.94 ± 0.00 ^a^	3.07 ± 0.00 ^c^	4.60 ± 0.00 ^a^
**50% BDP**	1.089 ± 0.00 ^c^	2.18 ± 0.01 ^c^	2.51 ± 0.01 ^f^	3.54 ± 0.00 ^d^
**100% BDP**	1.089 ± 0.00 ^c^	1.98 ± 0.00 ^c^	2.96 ± 0.01 ^d^	3.41 ± 0.00 ^e^
**50% wheat + 50% barley**	0.97 ± 0.03 ^d^	2.95 ± 0.01 ^a^	3.18 ± 0.00 ^bc^	4.06 ± 0.01 ^b^
**25% BDP**	0.97 ± 0.00 ^d^	2.74 ± 0.02 ^b^	2.99 ± 0.00 ^cd^	3.94 ± 0,00 ^b^
**50% BDP**	0.97 ± 0.01 ^d^	1.59 ± 0.00 ^d^	2.57 ± 0.02 ^ef^	3.70 ± 0.01 ^c^
**100% BDP**	0.96 ± 0.00 ^d^	1.42 ± 0.02 ^e^	2.63 ± 0.00 ^e^	3.04 ± 0.00 ^f^
**Samples**		***		
**Storage**		***		
**Samples × Storage**		***		

The mean ± SD of three replicates is used to represent the data. Duncan’s test at *p* < 0.05 indicates that the various letters denote the significance within each column. *** Highly significant difference at *p* < 0.05.

**Table 5:** Iron content of samples of biscuits made from wheat flour alone or mixed with barley flour and fortified with bisr date powder in different proportions.

Treatment	Iron Content (mg/100 g)
**Bisr date powder (BDP)**	5.14 ± 0.02
**100% wheat**	2.37 ± 0.01 ^f^
**25% BDP**	5.56 ± 0.00 ^e^
**50% BDP**	6.75 ± 0.02 ^d^
**100% BDP**	7.72 ± 0.00 ^c^
**50% wheat + 50% barley**	5.17 ± 0.03 ^e^
**25% BDP**	7.43 ± 0.00 ^c^
**50% BDP**	8.31 ± 0.00 ^b^
**100% BDP**	9.36 ± 0.00 ^a^

The mean ± SD of three replicates is used to represent the data. Duncan’s test at *p* < 0.05 indicates that the various letters denote the significance within each column.

**Table 6:** Sensory characteristics of biscuit samples made from wheat flour alone or mixed with barley flour and fortified with bisr date powder in different proportions.

Treatment	Color	Odor	Taste	Flavor	Overall Acceptability
**100% wheat**	7.67 ± 0.1 ^f^	8.43 ± 0.1 ^e^	7.83 ± 0.1 ^d^	7.79 ± 0.1 ^g^	8.08 ± 0.1 ^d^
**25% BDP**	7.79 ± 0.1 ^e^	8.52 ± 0.1 ^e^	8.70 ± 0.1 ^b^	8.00 ± 0.2 ^f^	8.80 ± 0.1 ^a^
**50% BDP**	8.67 ± 0.2 ^c^	8.80 ± 0.1 ^d^	8.85 ± 0.1 ^a^	8.17 ± 0.2 ^e^	8.33 ± 0.1 ^b c^
**100% BDP**	9.10 ± 0.2 ^b^	9.50 ± 0.2 ^b^	7.57 ± 0.1 ^e^	9.00 ± 0.2 ^b^	7.93 ± 0.1 ^e^
**50% wheat + 50% barley**	7.92 ± 0.1 ^e^	8.50 ± 0.1 ^e^	7.72 ± 0.1 ^d^	8.08 ± 0.1 ^f^	8.42 ± 0.2 ^b^
**25% BDP**	8.17 ± 0.1 ^d^	8.75 ± 0.1 ^d^	8.42 ± 0.2 ^c^	8.42 ± 0.1 ^d^	8.74 ± 0.1 ^a^
**50% BDP**	8.70 ± 0.1 ^c^	8.92 ± 0.2 ^c^	8.33 ± 0.2 ^c^	8.58 ± 0.1 ^c^	8.27 ± 0.2 ^c^
**100% BDP**	9.60 ± 0.2 ^a^	9.80 ± 0.2 ^a^	7.37 ± 0.2 ^f^	9.30 ± 0.2 ^a^	7.65 ± 0.1 ^f^

The mean ± SD of three replicates is used to represent the data. Duncan’s test at *p* < 0.05 indicates that the various letters denote the significance within each column.

**Table 7:** The physical nature of samples of biscuits made from wheat flour alone or mixed with barley flour and fortified with bisr date powder in different proportions.

Treatment	Diameter (cm)	Height (cm)	Spread Ratio (%)
**100% wheat**	5.08 ± 0.01 ^f^	0.53 ± 0.01 ^e^	9.60 ± 0.02 ^f^
**25% BDP**	5.62 ± 0.00 ^de^	0.57 ± 0.01 ^d^	9.85 ± 0.02 ^e^
**50% BDP**	5.78 ± 0.01 ^d^	0.59 ± 0.01 ^c^	9.94 ± 0.03 ^d^
**100% BDP**	6.22 ± 0.02 ^c^	0.62 ± 0.01 ^b^	10.03 ± 0.03 ^c^
**50% wheat + 50% barley**	5.54 ± 0.02 ^e^	0.56 ± 0.01 ^d^	9.90 ± 0.03 ^de^
**25% BDP**	6.12 ± 0.02 ^c^	0.59 ± 0.01 ^c^	10.37 ± 0.03 ^b^
**50% BDP**	6.58 ± 0.02 ^b^	0.63 ± 0.01 ^b^	10.46 ± 0.03 ^b^
**100% BDP**	7.81 ± 0.03 ^a^	0.72 ± 0.01 ^a^	10.86 ± 0.03 ^a^

The mean ± SD of three replicates is used to represent the data. Duncan’s test at *p* < 0.05 indicates that the various letters denote the significance within each column.

## Data Availability

The original contributions presented in the study are included in the article; further inquiries can be directed to the corresponding authors.
